# Disrupted fetal carbohydrate metabolism in children with autism spectrum disorder

**DOI:** 10.1186/s11689-025-09601-z

**Published:** 2025-03-29

**Authors:** Serena B. Gumusoglu, Brandon M. Schickling, Donna A. Santillan, Lynn M. Teesch, Mark K. Santillan

**Affiliations:** 1 Department of Obstetrics and Gynecology, University of Iowa, Iowa City, USA; 2 Iowa’s Hawkeye Intellectual and Developmental Disabilities Research Center (Hawk-IDDRC), Iowa City, USA; 3 Department of Chemistry, University of Iowa, Iowa City, USA

**Keywords:** Autism, Metabolomics, Carbohydrates, Monosaccharides, Polysaccharides, Cord blood

## Abstract

**Background:**

Despite the power and promise of early detection and treatment in autism spectrum disorder (ASD), early-life biomarkers are limited. An early-life risk biosignature would advance the field’s understanding of ASD pathogenies and targets for early diagnosis and intervention. We therefore sought to add to the growing ASD biomarker literature and evaluate whether fetal metabolomics are altered in idiopathic ASD.

**Methods:**

Banked cord blood plasma samples (*N* = 36 control, 16 ASD) were analyzed via gas chromatography and mass spectrometry (GC-MS). Samples were from babies later diagnosed with idiopathic ASD (non-familial, non-syndromic) or matched, neurotypical controls. Metabolite set enrichment analysis (MSEA) and biomarker prediction were performed (MetaboAnalyst).

**Results:**

We detected 76 metabolites in all samples. Of these, 20 metabolites differed significantly between groups: 10 increased and 10 decreased in ASD samples relative to neurotypical controls (*p* < 0.05). MSEA revealed significant changes in metabolic pathways related to carbohydrate metabolism and glycemic control. Untargeted principle components analysis of all metabolites did not reveal group differences, while targeted biomarker assessment (using only Fructose 6-phosphate, D-Mannose, and D-Fructose) by a Random Forest algorithm generated an area under the curve (AUC) = 0.766 (95% CI: 0.612–0.896) for ASD prediction.

**Conclusions:**

Despite a high and increasing prevalence, ASD has no definitive biomarkers or available treatments for its core symptoms. ASD’s earliest developmental antecedents remain unclear. We find that fetal plasma metabolomics differ with child ASD status, in particular invoking altered carbohydrate metabolism. While prior clinical and preclinical work has linked carbohydrate metabolism to ASD, no prior fetal studies have reported these disruptions in neonates or fetuses who go on to be diagnosed with ASD. Future work will investigate concordance with maternal metabolomics to determine maternal-fetal mechanisms.

**Supplementary Information:**

The online version contains supplementary material available at 10.1186/s11689-025-09601-z.

## Background

Autism spectrum disorder (ASD) is a significant and growing concern in the field of pediatrics. The American Academy of Pediatrics recommends developmental surveillance at every health supervision visit. This includes screening for ASD during 18- and 24-month well-child visits and standardized developmental screening at 9, 18, and 30 months of age [1]. Despite this high frequency of screening in early life, the average age of ASD diagnosis remains 4–5 years of age, indicating gaps in early detection.

Early detection and intervention have been robustly shown to improve long-term outcomes in individuals with neurodevelopmental disorders including ASD, though these efforts are stymied by a lack of operational biomarkers [2]. While there is widespread acknowledgment of the power and promise of such biomarkers [3, 4], few studies to date have examined biomarkers of an eventual ASD diagnosis at the earliest possible timepoint: during prenatal life. Prenatal next-generation sequencing (NGS) and whole exome sequencing, including fetal cell free RNA and DNA assay approaches, have improved the detection of genetic anomalies linked to developmental disorders, including ASD, allowing for early and precise diagnosis and ASD gene discovery [5]. Epigenomic studies have also emerged as an area of promise: one recent study in two high-familial risk prospective cohorts found sex-specific DNA methylation differences in the cord blood of ASD and typically developing children which may reflect differences in fetal brain transcription [6, 7]. In one of these cohorts, investigators further revealed genome-wide maternal blood transcriptomic changes associated with child ASD, honing in on six transcripts and co-expressed gene modules related to metabolite status and immune, histone modification, and RNA processing functions [8]. Finally, proteomic and metabolomic studies have provided important insights into ASD-linked environmental exposures. Recent studies have reported alterations to the placental proteome in a high-familial risk cohort (MARBLES) which are also linked to fetal brain development and ASD [9], revealing a potential for mid-gestation maternal metabolomics to bridge established maternal immune and environmental risk factor mechanisms in ASD [10]. Beyond improving early ASD detection and diagnosis, such early biomarkers have the potential to reveal early mechanisms of ASD pathogenesis, which remain largely unclear.

Work in recent decades demonstrates that ASD pathoetiology involves a combination of environmental and genetic factors [11]. Many environmental exposures begin *in utero* and include pesticide exposure, inflammatory insults, diseases of pregnancy (e.g., preeclampsia, gestational diabetes), infection, stress, maternal diet, and others. Further evidence suggests that environmental factors interact with genetic traits in fetal brain development to increase ASD risk [12]. The role of the “first,” *in utero* environment in driving ASD risk remains understudied.

Given that environmental contributors to ASD are poorly understood, the field requires sensitive, high-throughput, agnostic approaches for the study of environmental exposures or the “exposome.” Metabolomics is one such tool, quantifying metabolites, which are small molecule (< 1.5 kDA) substrates, intermediates, or products of cellular metabolism. In an era of multi-omics, metabolomics offers the advantages of being more sensitive to biological changes than other approaches. Gene and protein expression changes are amplified at the metabolome level (Lankadurai et al., 2013). Metabolomics is also more predictive of health status than isolated biomarkers [13]. Ultimately, metabolomics offers the advantage of providing a final read-out of interactions between nucleic acids and proteins.

Here, we assessed cord blood metabolomic differences between idiopathic ASD and control pregnancies. We hypothesized that differences in cord blood metabolomics in this ASD population might reveal environmental mechanisms of prenatal ASD programming. Our results reveal altered levels of mono and polysaccharide metabolism and decreased branched chain amino acid metabolism (keto acids) in ASD samples, suggesting a role for altered carbohydrate metabolism in prenatal ASD programming.

## Methods

### Participants

A total of 52 cord blood samples were obtained from the University of Iowa Perinatal Family Tissue Bank (IRB: 200910784): 16 from pregnancies resulting in a child later diagnosed with ASD and 36 from pregnancies resulting in a child without any documented mental, behavioral, or neurodevelopmental disorder diagnosis. At the time of diagnosis, all children were between the ages of 2 and 12. Neurotypical controls were confirmed to have no neurodevelopmental diagnoses at between 4 and 12 years of age. ASD group inclusion criteria included a clinician diagnosis of Autistic disorder (ICD-10 code F84.0 or similar documentation). Extremely premature (< 28 weeks gestation) and extremely low birthweight (< 1,000 g) infants were excluded.

### Clinical data acquisition

To interrogate clinical data which correspond to biospecimens analyzed here, the University of Iowa Intergenerational Health Knowledgebase (IHK) was utilized. The IHK is a clinical research data warehouse which contains information from the electronic health record (IRB: 202101369) [14]. Clinical data from the IHK (e.g., diagnostic codes) can be linked via an anonymous identifier to PFTB samples [14]. ASD diagnoses were further manually verified by a study author with expertise in neurodevelopmental disorders (SBG).

### Sample collection and processing

After obtaining informed consent for participation in the University of Iowa Perinatal Family Tissue Bank, umbilical cord blood samples were collected, processed, and stored as described previously [15]. Briefly, all samples were collected immediately after delivery of the placenta into evacuated Citrate Phosphate Dextrose Solution tubes (Fenwal Technologies). Cord blood was collected at the bedside. After collection, samples were processed for plasma, which was aliquoted, snap frozen, and stored at − 80 °C for subsequent metabolomic profiling.

### Metabolite extraction

To process banked cord blood samples for metabolite extraction, plasma samples were first diluted in 18 volumes of ice-cold methanol, acetonitrile, and water (2:2:1 mixture) containing internal standards (D4-citric acid, D4-succinic acid, D8-valine, and U13C-labeled glutamine, glutamic acid, lysine, methionine, serine, tryptophan; all from Cambridge Isotope Laboratories). These parameters have been shown to be well-suited to plasma metabolite extraction [16, 17].

Next, a volume equivalent to the plasma volume was removed from the water component of the extraction buffer. The resulting extraction mixture was then vortexed at room temperature for 10 min, rotated for 12 h at -20 °C, and centrifuged at 21,000*g* for 10 min. Finally, 75 µl of cleared metabolite extract was transferred to an autosampler vial and dried using a SpeedVac vacuum container (Thermo).

After drying, metabolite extracts were reconstituted in 30 µl of 11.4 mg/ml methoxyamine (MOX) in anhydrous pyridine. This was vortexed for 5 min then heated to 60 °C for 1 h before 20 µl of N, O-Bis(trimethylsilyl)trifluoroacetamide (TMS) was added to each sample. This final mixture was then vortexed for 1 min and heated to 60 °C for 30 min.

### Gas chromatography–mass spectrometry

The analysis of derivatized samples was performed using gas chromatography–mass spectrometry (GC-MS), as previously [17]. A 1 µl volume of the derivatized sample was introduced into a Trace 1300 GC system (Thermo) equipped with a TraceGold TG-5SilMS column (Thermo). The GC system was operated under specific conditions, including a split ratio of 5:1, a split flow rate of 24 µl/minute, a purge flow rate of 5 ml/minute, and a carrier mode set to Constant Flow with a carrier flow rate of 1.2 ml/minute. Separation was carried out using a standard fused silica TraceGold TG-5SilMS column (Thermo).

The temperature gradient in the GC oven was programmed as follows: initiated and maintained at 80 °C for 3 min, after which it increased at a rate of 20 °C per minute until reaching 280 °C, and then finally held at 280 °C for 8 min.

For ion detection, an ISQ 7000 mass spectrometer by Thermo was utilized in electron ionization (EI) mode with an energy of -70 eV over a time range of 3.90–21.00 min. Detection employed select ion monitoring. In total, 76 total metabolites were detected in both groups and across all samples (Supplementary Table 1).

### Statistical analyses

TraceFinder 5.1 software (Thermo) was utilized to analyze raw data. The process of identifying and annotating metabolites involved several criteria: a minimum requirement of two ions (comprising target and confirmation ions), as well as a unique retention time that matched the ions and the retention time of a previously established in-house reference standard. The NOREVA tool was used to ensure accurate peak intensities. Samples were pooled and analyzed before derivatization at the outset, during, and at the end of the analytical run. After NOREVA correction, data underwent ratiometric normalization using TRN normalization. Data were uniformly scaled for visualization purposes. The Grubbs’ test (α = 0.01) identified outliers; none were found.

During autoscaling, analyte levels for each participant were centered relative to their respective mean values and divided by the standard deviation of each analyte. A normal distribution was confirmed. Both univariate hypothesis testing and multivariate orthogonal partial least-squares (OPLS) modeling, as previously described [18], were employed. T-tests or chi-square tests were used to assess group differences, as appropriate. Metabolite set enrichment analyses (MSEA) identified biologically meaningful patterns within the dataset, as outlined previously [19]. MSEA analysis can contextualize biomarker features and reveal both shared effects and potential new biomarkers, enhancing metabolic disorder diagnostics [20]. Briefly, enrichment tests utilized a generalized linear model to compute a Q statistic, which represents the average covariance between metabolites and the outcome of interest (ASD vs. neurotypical controls). Enrichment P values were calculated for enrichment of the sample set versus a reference metabolome containing 73 (of 76 total detected) catalogued metabolites. Only sets containing at least two metabolites were considered enriched.

All statistical comparisons were performed using MetaboAnalyst 5.0 [18]. Figures were generated in or adapted from MetaboAnalyst 5.0. Continuous variables are presented as mean ± SEM and were compared using Student’s t-tests, as is standard in the field [21–23]. Categorical variables are expressed as percentages and were assessed using Chi-square analysis. *P* < 0.05 was defined as statistically significant.

## Results

### Cohort characteristics

A summary of cohort demographics and clinical characteristics are shown in Table 1, comparing pregnancies resulting in a child with a later ASD diagnosis (“ASD pregnancies”) to those resulting in a neurotypical control child (“Neurotypical pregnancies”). Demographics (sex, ethnicity), maternal characteristics [maternal age, delivery type, gravida, parity, body mass index (BMI) at new OB visit], and delivery outcomes (gestational age, birth weight, Apgar scores at 1 and 5 min) did not differ between groups. Only infant sex was significantly different between neurotypical controls and ASD pregnancies—male infants were more common among ASD pregnancies (77%) than neurotypical pregnancies (42%) (*p* = 0.03). This is representative of the typical male bias in ASD [24, 25]. In the ASD group, two mothers had type 1 diabetes while in the control group one had gestational diabetes and one had type 2 diabetes.

**Table 1:** Cohort characteristics

	Neurotypical (*n* = 36)	ASD (*n* = 16)	*P*-value
BMI at New OB visit (kg/m^2^)	33.43 ± 10.21	30.38 ± 6.05	0.38
Gestational age (weeks)	38.33 ± 1.43	38.51 ± 1.81	0.73
Birth Weight (g)	3275.11 ± 497.05	3501 ± 816.41	0.29
Ethnicity (% Hispanic or Latino)	3%	14%	0.14
Race (% non-white)	3%	21%	0.06
Infant Sex (% male)	42%	77%	**0.03**
Maternal age (years)	28.18 ± 8.15	24.89 ± 10.62	0.23
APGAR at 1 min (median)	8.00 ± 1.67	8.78 ± 0.44	0.18
APGAR at 5 min (median)	8.67 ± 0.76	8.93 ± 0.27	0.22
Cesarean Delivery (%)	25%	29%	0.80
Gravida	3.03 ± 1.93	3.3 ± 1.95	0.70
Parity	1.42 ± 1.23	1.40 ± 1.71	0.96

Cord blood samples were analyzed from pregnancies resulting in a child without any diagnosed neurodevelopmental disorder (neurotypical) or with Autism Spectrum Disorder (ASD). Only male sex was significantly different between groups (more prevalent in ASD). Values reflect mean ± standard deviation unless otherwise noted. Categorical variables were compared via chi-square. Continuous variables were compared via Student’s t-test. *P* < 0.05 was significant. Bolded findings are statistically significant

### Metabolomic differences between ASD and neurotypical pregnancies

**Fig. 1:**
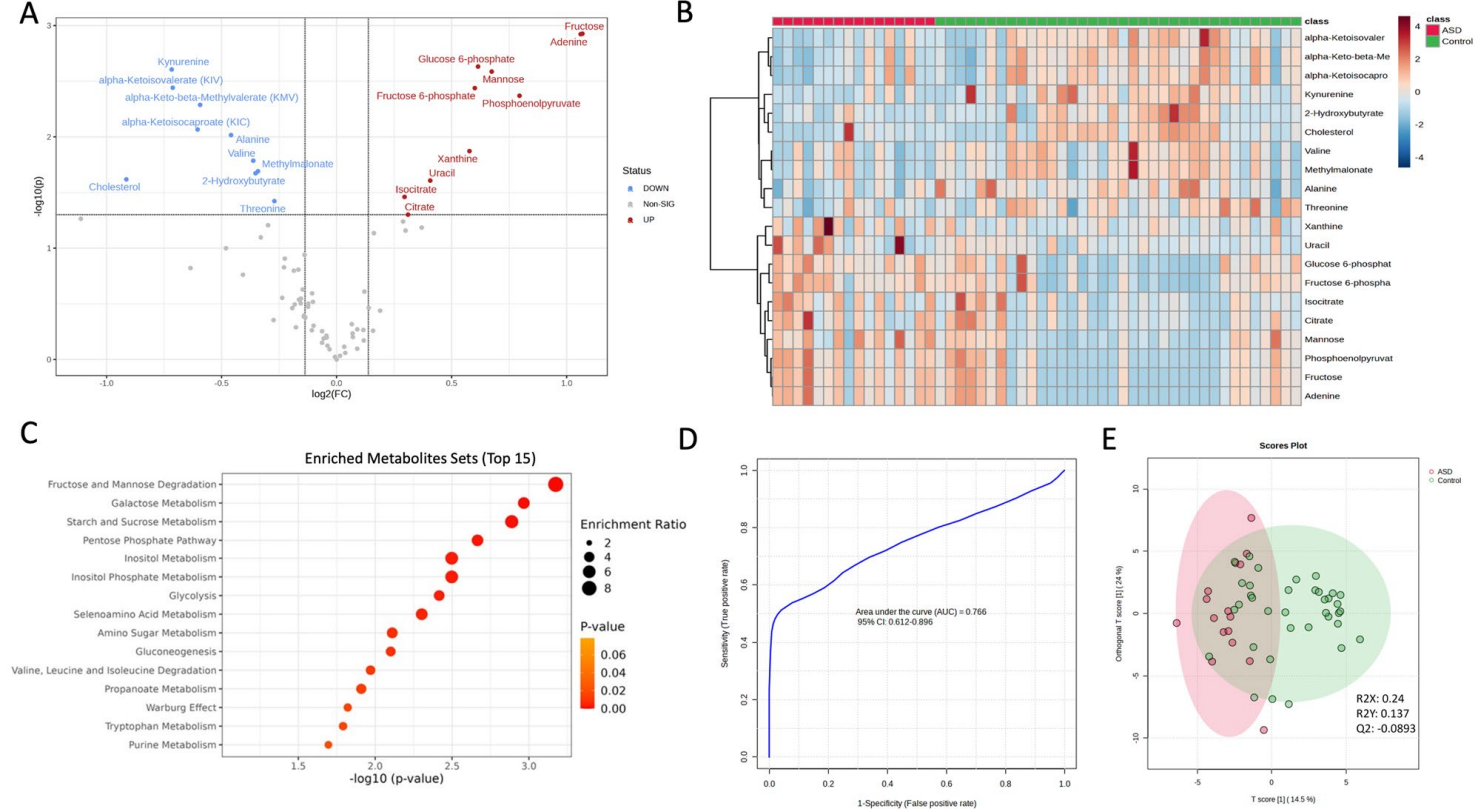
Comparison of metabolites in neurotypical versus ASD cord blood. **A**) Volcano plot depicting significantly increased and decreased metabolites in cord blood of neurotypical versus ASD pregnancies. P value threshold = 0.05 (by Student’s t-tests); fold change threshold = 1.1 (ASD/Control) to allow for comprehensive exploration of the data. **B**) Hierarchical clustering heatmap depicting the top 20 (by t-test) significantly changed metabolites in neurotypical versus ASD pregnancies. Data are normalized, autoscaled, and Euclidean distances measured. Samples are clustered by the Ward method. **C**) Metabolite set enrichment analysis (MSEA) of ASD versus neurotypical control pregnancies reveals enrichment of select metabolic sets. Analyses utilized built-in MSEA library (99 entries). Only the top 15 significantly enriched metabolite sets containing more than one metabolite are displayed. Significant enrichment (*P* < 0.05) was determined relative to a reference metabolome containing 73 detected metabolites. **D**) Receiver operating characteristic (ROC) analysis was built on select features to predict ASD pregnancy. Features were those identified in the top-most significantly enriched metabolite set by GSEA (fructose and mannose degradation): Fructose 6-phosphate, D-Mannose, and D-Fructose. The area under the curve (AUC) was generated as the average of 100 cross validations utilizing a random forest algorithm. **E**) Scores plot of multivariate modeling by the orthogonal partial least-squares (OPLS) approach reveals highlight overlapping overall metabolome between ASD and neurotypical control pregnancies. Score plot depicts percentage of response variable explained by the first predictor only (T score[1]). R2 (percent variance explained by predictor) and Q2 (cross-validation) quality metrics are listed on the figure and indicate over-fitting. Variables were standardized (mean-centered, unit-variance scaled) prior to modeling. Ellipses correspond to 95% of the multivariate normal distribution with sample covariances for each class. Figures modified from MetaboAnalyst

In ASD pregnancies relative to Neurotypical pregnancies, 20 metabolites were significantly changed in the cord blood (Fig. 1A). Of these, 10 were significantly increased, while 10 were significantly decreased (Table 2). Given sex differences between the two cohorts, the analyses were rerun within controls to determine a baseline sex effect, which did not overlap with ASD-related metabolite differences.

**Table 2:** Analyses of ASD and neurotypical control pregnancy cord blood metabolites reveal significant metabolite changes

	Fold change (FC)	log2(FC)	*P* value
Fructose	2.097	1.068	0.0012
Adenine	2.086	1.061	0.0012
Glucose 6-phosphate	1.531	0.615	0.0023
Kynurenine	0.608	-0.717	0.0025
Mannose	1.596	0.674	0.0026
alpha-Ketoisovalerate (KIV)	0.610	-0.712	0.0036
Fructose 6-phosphate	1.516	0.601	0.0036
Phosphoenolpyruvate	1.735	0.795	0.0043
alpha-Keto-beta-Methylvalerate (KMV)	0.663	-0.593	0.0052
alpha-Ketoisocaproate (KIC)	0.658	-0.604	0.0086
Alanine	0.728	-0.459	0.0096
Xanthine	1.492	0.577	0.0134
Valine	0.778	-0.362	0.0164
Methylmalonate	0.789	-0.342	0.0204
2-Hydroxybutyrate	0.783	-0.352	0.0212
Cholesterol	0.531	-0.914	0.0241
Uracil	1.326	0.407	0.0247
Isocitrate	1.227	0.295	0.0346
Threonine	0.829	-0.270	0.0377
Citrate	1.240	0.310	0.0499

Metabolites are ranked by P value. Comparisons made by student’s t-test (*P* < 0.05)

Hierarchical clustering was used to next generate a heatmap depicting these top 20 (by t-test) significantly changed metabolites in ASD and neurotypical control pregnancies (Fig. 1B). Hierarchical clustering revealed broad group differences by Euclidean distances.

### Metabolite set enrichment analysis of ASD and neurotypical pregnancies

Next, metabolite set enrichment analyses (MSEA) was completed to identify biologically meaningful patterns of disruption cord blood from ASD versus neurotypical control pregnancies (Fig. 1C). Sixteen sets were found to be significantly (*P* < 0.05) enriched (Table 3), with the top enriched sets by p-value being fructose and mannose degradation, galactose metabolism, and starch and sucrose metabolism. Some metabolites recurred across more than five sets: Glucose 6-phosphate occurred across eight, as did Pyruvic acid, while Fructose-6-phosphate recurred across 6 sets. The top-most over-represented set, Fructose and mannose degradation, included Fructose 6-phosphate, D-Mannose, and D-Fructose, which were represented across many sets. Glycolysis and Gluconeogenesis sets had largely overlapping metabolites (D-Glucose, Fructose 6-phosphate, Pyruvic acid, Phosphoenolpyruvic acid, 3-Phosphoglyceric acid, and Glucose 6-phosphate), and The Warburg Effect set overlapped with both (D-Glucose, Fructose 6-phosphate, Pyruvic acid, Phosphoenolpyruvic acid, 3-Phosphoglyceric acid, Glucose 6-phosphate). The Fructose and Mannose Degradation, Galactose Metabolism, and Starch and Sucrose Metabolism sets also shared five metabolites (D-Glucose, Fructose 6-phosphate, D-Mannose, D-Fructose, and Glucose 6-phosphate). Finally, while the two inositol-related sets (Inositol Metabolism and Inositol Phosphate Metabolism) shared 100% of their annotated metabolites (Glucose 6-phosphate and Inositol), only one of these metabolites (Glucose 6-phosphate) occurred in other sets.

**Table 3:** Metabolite set enrichment analyses (MSEA) revealed biologically meaningful metabolite sets disrupted in ASD pregnancies. sets containing at least 2 metabolites were significantly (*P* < 0.05) overrepresented by metabolites altered in cord blood of ASD versus neurotypical pregnancies. “Hits” describe overlapping metabolites between MSEA metabolite set and those differentially expressed between ASD and control samples. “Total set size” describes the total number of metabolites contained within each annotated set (Metaboanalyst)

Metabolite Set	Total Set Size	Hits	Q Statistic	*P* value
Fructose and Mannose Degradation	31	3	17.193	*0.0007*
Galactose Metabolism	38	6	9.9332	*0.0011*
Starch and Sucrose Metabolism	31	3	12.519	*0.0013*
Pentose Phosphate Pathway	29	4	9.6355	*0.0022*
Inositol Metabolism	30	2	11.853	*0.0032*
Inositol Phosphate Metabolism	24	2	11.853	*0.0032*
Glycolysis	23	6	8.23	*0.0038*
Selenoamino Acid Metabolism	27	2	9.9321	*0.0050*
Amino Sugar Metabolism	33	6	8.5245	*0.0078*
Gluconeogenesis	33	8	7.0914	*0.0079*
Valine, Leucine and Isoleucine Degradation	59	9	6.7813	*0.0107*
Propanoate Metabolism	42	5	7.526	*0.0123*
Warburg Effect	57	16	5.4267	*0.0151*
Tryptophan Metabolism	59	8	5.5524	*0.0162*
Purine Metabolism	73	9	4.9873	*0.0202*
Steroid Biosynthesis	43	2	6.2884	*0.0391*

Sets were significantly (*P* < 0.05) overrepresented by metabolites altered in cord blood of ASD versus neurotypical pregnancies

### Receiver operating characteristic curve analysis on selected features reveals a potential cord blood ASD biomarker while principal components analysis reveals no metabolome-wide difference

A targeted receiver operating characteristic (ROC) analysis was performed on features in the top-most significantly enriched metabolite set by GSEA: fructose and mannose degradation. This contained three metabolite hits: Fructose 6-phosphate, D-Mannose, and D-Fructose. The area under the curve (AUC), generated via the average of 100 cross validations utilizing a random forest algorithm relying only on Fructose 6-phosphate, D-Mannose, and D-Fructose, was 0.766 (95% CI: 0.612–0.896) (Fig. 1D).

Unlike targeted ROC analysis, untargeted multivariate modeling by orthogonal partial least-squares (OPLS) revealed no dataset-wide distinction between ASD and neurotypical pregnancy metabolomics (Fig. 1E). This approach provides a single model for the entirety of the metabolomics dataset and efficiently handles multicollinear chemometric predictors. OPLS was selected given its similar predictive capacity to other PLS approaches despite requiring only one predictive component [26]. Separation between ASD and control pregnancies was poor with this approach, with a high degree over overlap between the multivariate normal distribution of each class. Q2Y metrics revealed overfitting of the model (Q2: -0.0893).

## Discussion

Here, we describe one of few published analyses of cord blood metabolomics in ASD. Our use of a large, thoroughly annotated, longitudinal biobank allowed us to examine early-life alterations in feto-placental metabolomic milieu with confidence in eventual child diagnosis and other characteristics. We find that changes in fructose, mannose, galactose, starch and sucrose, and other sugar metabolism pathways are enriched in the metabolomics of ASD cord blood plasma. Furthermore, machine learning classifiers using only the top-most enriched metabolites (Fructose 6-phosphate, D-Mannose, and D-Fructose) distinguished ASD cases from neurotypical controls well, with an area under the receiver operating characteristic (ROC) value of 0.766.

Our findings strongly implicate altered Mannose and Fructose (Fructose 6-phosphate, D-Mannose, and D-Fructose all increased in ASD samples) and their metabolism. By metabolite set enrichment analyses, “fructose and mannose degradation” was the most disrupted metabolite set in ASD pregnancies, and a number of additional sets reflected similar changes in sugar metabolism (e.g., fructose and glucose overlap between glycolysis, gluconeogenesis, pentose phosphate pathway, starch and sucrose metabolism, and amino sugar metabolism pathways). The literature offers some context for this key finding: disorders of glucose and fructose metabolism (e.g., diabetes), as well as diets enriched for these sugars, are linked to neurodevelopmental disorders including ASD. For example, a 2015 study of more than 322,000 children in California found that early gestational diabetes (before 26 weeks) raised autism risk by 42% [27]. Another study found a positive association between maternal Western diet, which is high in sugar, and child ASD (ß=11.19, 95% CI: 3.30–19.90), though this correlation was attenuated by adjustment for total energy intake [28]. Preclinical work also suggests that a high-sugar maternal diet causes deficits in rat offspring which are suggestive of ASD-like pathology, such as impaired behavioral flexibility and redox stress in offspring brain [29]. Abnormal fructose metabolism is implicated in cell signaling and oxidative stress mechanisms, as well as inflammation [30]. Studies of maternal high sugar diet implicate neuroinflammatory and oxidative stress mechanisms in offspring neuroprogramming, though additional mechanistic work is needed to more precisely clarify mediators and therapeutic targets [31].

Metabolomics in pregnancy is an emerging and promising frontier [32]. Amniotic fluid metabolomic biomarkers boast accuracy as high as 96.3% for the prediction of preterm labor [33], with another study reporting a mean predictive accuracy of 90% and false negative rate of 14.2% for preterm birth [33].

Cord blood metabolomics allows for proximal measures of the fetal metabolome, which may offer insights into the programming of developmental disease. While some results have been mixed [34, 35], recent studies have found that cord blood metabolomics are altered relative to maternal and fetal/infant characteristics, for example with maternal weight gain in pregnancy and obesity [36, 37], infant birth weight [38, 39], preeclampsia [40], infant allergy and asthma [41, 42], intrauterine hypergylcemia [43], intrauterine growth restriction [44–47], perinatal asphyxia [47] and hypoxic-ischemic encephalopathy [46, 48–51], neonatal macrosomia [52], polychlorinated biphenyl exposure [53], diabetes [54], and cesarean delivery [55]. Despite this prior work, limited studies have focused on cord blood metabolomics and neurodevelopmental outcomes such as ASD.

In the present study, we tested cord blood plasma metabolites for differences between newborns later diagnosed with idiopathic ASD and typically developing controls. Our study suggests that changes in carbohydrate metabolites may be mechanistic harbingers of ASD risk, which recent work has found are driven by functional gut microbiome architecture. Feto-placental carbohydrate metabolism is tightly regulated and is dependent on the balance between exogenous glucose from maternal circulation and fetal glucose and lactate utilization [56]. In neonatal life, endogenous glucose production and intake rapidly supplant placental sources to the infant. Fetal growth, which is altered in some subsets of ASD [57], is tightly correlated with placental carbohydrate metabolism. Umbilical cord leukocytes from fetal inflammatory response syndrome cases exhibit dysregulated carbohydrate metabolism machinery [58], further demonstrating a role for immune-metabolism interactions in these changes. Immune activation in fetal life is a long-studied risk factor for ASD [59].

Few prior studies have assessed metabolites in ASD cord blood. One recent, untargeted metabolomics study of the Norwegian Autism Birth Cohort (*n* = 418) reported changes in sugar alcohols in cord blood from male but not female ASD pregnancies, and increased glucose-6-phospate in maternal blood from male ASD pregnancies [60]. We report increased glucose-6-phosphate in ASD cord blood plasma. This prior work also reports increased predictive performance of cord blood versus maternal blood metabolomics for distinguishing ASD from neurotypical control samples.

Another recent study in the Markers of Autism risk in Babies: Learning Early Signs (MARBLES) cohort (*n* = 142 cord blood plasma samples) reported untargeted metabolomics in a high-risk population. Mothers in MARBLES are selected on the basis of already having a child with an ASD diagnosis, thereby loading this sample for increased genetic risk. This study revealed no significant negative or positive ASD predictive value of the cord blood metabolomics feature set [61]. Not the Norwegian Patient Registry [60] study, nor MARBLES, had the same explicit focus on non-genetic or idiopathic cases as our study, which may explain divergent findings. Genetic and syndromic ASD may not reflect the same metabolic milieu as the idiopathic cases we utilized here.

Use of cord blood plasma in the present study is an advantage given it is reflective of placental, maternal, and fetal biologic processes. Particularly in the biomarker discovery phase, it is advantageous to select a sample closest to the disease process [62]. Future studies should validate our findings in placenta and in maternal plasma, which is easier to obtain and may be evaluated early in pregnancy. Differences between cord blood, placenta, and maternal plasma metabolomes may also reflect fundamental metabolic processes in the feto-placental unit and yield additional hypotheses about the metabolomics of ASD in early life [32]. Amniotic fluid is another potential target for studying fetal metabolomics. Amniotic fluid metabolomics are highly predictive of fetal malformation and prematurity [33, 63], and may further reflect metabolic processes in fetal life.

Our studies have additional implications for research on the exposome in early life. Children’s health may benefit from more integrated use of biosampling and biomarker detection studies, including in the newborn period [64]. The cord blood is a particularly attractive sample because it reflects fetal, placental, and maternal exposures. Prior cord blood metabolomics studies have previously centered on neonatal complications following delivery [65]. Longitudinal studies may also identify critical exposures and vulnerable periods that have lifelong impacts on individual well-being. The power of this approach for understanding long-term programming of neurodevelopment, metabolism, cardiovascular disease, and other health processes remains mostly untapped.

Our use of highly standardized and timely biospecimen collection and storage processes through the University of Iowa Perinatal Family Tissue Bank is a distinct advantage of the present study [15]. This active tissue bank contains samples from over 6,000 pregnancies collected continuously over more than 10 years. Samples collected with standardized protocols reflect mixed arterial and veinous origins, which somewhat limits interpretation of fetal versus placental contributions. Studies find subtle but significant differences in umbilical cord venous and arterial plasma metabolites, with the main differences related to amino acid and energy metabolism [66]. Differences between arterial and venous supplies should be dissected in future work.

A limitation of the present study and of metabolomics more broadly is that plasma and other biofluid metabolomics are highly dynamic and reflect metabolite status at only one moment in time. Outputs are impacted by immune and dietary conditions, for example. Isolated samples from the end of pregnancy are therefore not reflective of longer-term dynamics throughout gestation. Our evaluation of only a subset of curated, targeted metabolites is a significant limitation, as are the small sample size and lack of a validation cohort. Larger validation studies in more diverse populations are the focus of our future and ongoing work. Additionally, our GC-MS metabolomic findings should be validated or expanded using alternative methods. For example, liquid chromatography-mass spectrometry (LC-MS) may be particularly useful for analyzing a broader range of metabolites than GC-MS, including larger molecules such as peptides and proteins present in biofluids such as urine [67]. Nuclear magnetic resonance (NMR) spectroscopy offers high reproducibility and the ability to analyze complex mixtures such as serum [68], though it has lower sensitivity than mass spectrometry-based methods [69]. While GC-MS was selected here given its high sensitivity and specificity for use in biofluids and complex biological matrices [70], as well as high degree of standardized (> 50 years of established protocols) [71], other methods should also be considered in future work.

Despite limitations, the present study elucidates a potential role for altered carbohydrate metabolism in the pathogenesis of idiopathic autism spectrum disorder. Our use of an idiopathic, non-syndromic or familiar cohort allowed for a particular focus on potential environmental drivers of ASD pathogenesis. Additionally, while prior work has focused on isolated biomarkers in ASD pathogenesis, our focus was instead on pathways and networks of metabolites. Untargeted, multi-omics strategies, for example coupling metabolomics with transcriptomics and genomics, may further improve movement towards a systems biology approach towards prenatal and obstetrics diagnostics [13, 72]. A wholistic approach may improve understandings of how developmental programming mechanisms shape genetic-by-environmental interactions in ASD pathogenesis. Integration strategies using machine learning for disease modeling and classification provide another, complimentary opportunity for advance [73, 74]. Our study demonstrates the potential power of machine learning approaches using only select, mechanistically-informed features. This has implications for the advancement of early screening and detection of pediatric disorders broadly.

## Conclusions

ASD is one of the most prevalent and fastest growing neurodevelopmental disorders, impacting pediatric patients and their providers in a multitude of ways. Studies such as this one, which reveal early-life processes in ASD pathogenesis and fetal origins, as well as potential biomarkers to improve ASD detection, offer hope for improving future diagnostic, treatment, and prevention strategies. To improve outcomes for children and their families, it is imperative that the field move towards early detection and diagnosis empowered by robust biomarkers.

## Electronic supplementary material

Below is the link to the electronic supplementary material.

Supplementary Material 1

## Data Availability

Data are available upon request.

## Abbreviations

ASD: Autism spectrum disorder
GC-MS: Gas chromatography–mass spectrometry
MSEA: Metabolite set enrichment analysis
IHK: Intergenerational Health Knowledgebase
ROC: Receiver operating characteristic
OPLS: Orthogonal partial least-squares
MARBLES: Markers of Autism risk in Babies: Learning Early Signs

## References

[1] Lipkin PH, Macias MM, Council On Children With Disabilities SOD, Behavioral P. Promoting optimal development: identifying infants and young children with developmental disorders through developmental surveillance and screening. Pediatrics. 2020;145(1). 10.1542/peds.2019-3449 31843861

[2] Campbell F, Conti G, Heckman JJ, Moon SH, Pinto R, Pungello E, et al. Early childhood investments substantially boost adult health. Science. 2014;343(6178):1478–85. 24675955 10.1126/science.1248429 PMC4028126

[3] Anderson GM. Autism biomarkers: challenges, pitfalls and possibilities. J Autism Dev Disord. 2015;45(4):1103–13. 25193140 10.1007/s10803-014-2225-4

[4] Bristol-Power MM, Spinella G. Research on screening and diagnosis in autism: a work in progress. J Autism Dev Disord. 1999;29(6):435–8. 10638458 10.1023/a:1021991718423

[5] Sanders SJ. Next-Generation sequencing in autism spectrum disorder. Cold Spring Harb Perspect Med. 2019;9(8). 10.1101/cshperspect.a026872 PMC6671934 30420340

[6] LaSalle JM. Epigenomic signatures reveal mechanistic clues and predictive markers for autism spectrum disorder. Mol Psychiatry. 2023;28(5):1890–901. 36650278 10.1038/s41380-022-01917-9 PMC10560404

[7] Mordaunt CE, Jianu JM, Laufer BI, Zhu Y, Hwang H, Dunaway KW, et al. Cord blood DNA methylome in newborns later diagnosed with autism spectrum disorder reflects early dysregulation of neurodevelopmental and X-linked genes. Genome Med. 2020;12(1):88. 33054850 10.1186/s13073-020-00785-8 PMC7559201

[8] Zhu Y, Mordaunt CE, Durbin-Johnson BP, Caudill MA, Malysheva OV, Miller JW, et al. Expression changes in epigenetic gene pathways associated with One-Carbon nutritional metabolites in maternal blood from pregnancies resulting in autism and Non-Typical neurodevelopment. Autism Res. 2021;14(1):11–28. 33159718 10.1002/aur.2428 PMC7894157

[9] Parenti M, Schmidt RJ, Ozonoff S, Shin HM, Tancredi DJ, Krakowiak P et al. Maternal serum and placental metabolomes in association with prenatal phthalate exposure and neurodevelopmental outcomes in the MARBLES cohort. Metabolites. 2022;12(9). 10.3390/metabo12090829 PMC9500898 36144233

[10] Ritz B, Yan Q, Uppal K, Liew Z, Cui X, Ling C, et al. Untargeted metabolomics screen of Mid-pregnancy maternal serum and autism in offspring. Autism Res. 2020;13(8):1258–69. 32496662 10.1002/aur.2311 PMC13292088

[11] Bai D, Yip BHK, Windham GC, Sourander A, Francis R, Yoffe R, et al. Association of genetic and environmental factors with autism in a 5-Country cohort. JAMA Psychiatry. 2019;76(10):1035–43. 31314057 10.1001/jamapsychiatry.2019.1411 PMC6646998

[12] Courchesne E, Pramparo T, Gazestani VH, Lombardo MV, Pierce K, Lewis NE. The ASD living biology: from cell proliferation to clinical phenotype. Mol Psychiatry. 2019;24(1):88–107. 29934544 10.1038/s41380-018-0056-y PMC6309606

[13] Horgan RP, Clancy OH, Myers JE, Baker PN. An overview of proteomic and metabolomic technologies and their application to pregnancy research. BJOG. 2009;116(2):173–81. 19076949 10.1111/j.1471-0528.2008.01997.x

[14] Santillan DA, Santillan MK, Davis HA, Crooks M, Flanagan PJ, Ortman CE et al. Implementation of a Maternal Child Knowledgebase. AMIA Annu Symp Proc. 2022;2022:432-8. PMC9285168 35854751

[15] Santillan MK, Leslie KK, Hamilton WS, Boese BJ, Ahuja M, Hunter SK, et al. Collection of a lifetime: a practical approach to developing a longitudinal collection of women’s healthcare biological samples. Eur J Obstet Gynecol Reprod Biol. 2014;179:94–9. 24965987 10.1016/j.ejogrb.2014.05.023 PMC4148073

[16] Lepoittevin M, Blancart-Remaury Q, Kerforne T, Pellerin L, Hauet T, Thuillier R. Comparison between 5 extractions methods in either plasma or serum to determine the optimal extraction and matrix combination for human metabolomics. Cell Mol Biol Lett. 2023;28(1):43. 37210499 10.1186/s11658-023-00452-x PMC10200056

[17] Zhang Y, Taufalele PV, Cochran JD, Robillard-Frayne I, Marx JM, Soto J, et al. Mitochondrial pyruvate carriers are required for myocardial stress adaptation. Nat Metab. 2020;2(11):1248–64. 33106689 10.1038/s42255-020-00288-1 PMC8015649

[18] Pang Z, Zhou G, Ewald J, Chang L, Hacariz O, Basu N, et al. Using metaboanalyst 5.0 for LC-HRMS spectra processing, multi-omics integration and covariate adjustment of global metabolomics data. Nat Protoc. 2022;17(8):1735–61. 35715522 10.1038/s41596-022-00710-w

[19] Lu Y, Pang Z, Xia J. Comprehensive investigation of pathway enrichment methods for functional interpretation of LC-MS global metabolomics data. Brief Bioinform. 2023;24(1). 10.1093/bib/bbac553 PMC9851290 36572652

[20] Hoegen B, Hampstead JE, Engelke UFH, Kulkarni P, Wevers RA, Brunner HG, et al. Application of metabolite set enrichment analysis on untargeted metabolomics data prioritises relevant pathways and detects novel biomarkers for inherited metabolic disorders. J Inherit Metab Dis. 2022;45(4):682–95. 35546254 10.1002/jimd.12522 PMC9544878

[21] Bartel J, Krumsiek J, Theis FJ. Statistical methods for the analysis of high-throughput metabolomics data. Comput Struct Biotechnol J. 2013;4:e201301009. 24688690 10.5936/csbj.201301009 PMC3962125

[22] Xi B, Gu H, Baniasadi H, Raftery D. Statistical analysis and modeling of mass spectrometry-based metabolomics data. Methods Mol Biol. 2014;1198:333–53. 25270940 10.1007/978-1-4939-1258-2_22 PMC4319703

[23] Moseley HN. Error analysis and propagation in metabolomics data analysis. Comput Struct Biotechnol J. 2013;4(5). 10.5936/csbj.201301006 PMC3647477 23667718

[24] Calderoni S. Sex/gender differences in children with autism spectrum disorder: A brief overview on epidemiology, symptom profile, and neuroanatomy. J Neurosci Res. 2023;101(5):739–50. 35043482 10.1002/jnr.25000

[25] Ferri SL, Abel T, Brodkin ES. Sex differences in autism spectrum disorder: a review. Curr Psychiatry Rep. 2018;20(2):9. 29504047 10.1007/s11920-018-0874-2 PMC6477922

[26] Thevenot EA, Roux A, Xu Y, Ezan E, Junot C. Analysis of the human adult urinary metabolome variations with age, body mass index, and gender by implementing a comprehensive workflow for univariate and OPLS statistical analyses. J Proteome Res. 2015;14(8):3322–35. 26088811 10.1021/acs.jproteome.5b00354

[27] Xiang AH. Association of maternal diabetes with autism in offspring. JAMA. 2017;317(5):537–8. 28170476 10.1001/jama.2016.20122

[28] Vecchione R, Wang S, Rando J, Chavarro JE, Croen LA, Fallin MD et al. Maternal dietary patterns during pregnancy and child Autism-Related traits: results from two US cohorts. Nutrients. 2022;14(13). 10.3390/nu14132729 PMC9268965 35807909

[29] Lopez-Taboada I, Sal-Sarria S, Vallejo G, Coto-Montes A, Conejo NM, Gonzalez-Pardo H. Sexual dimorphism in Spatial learning and brain metabolism after exposure to a Western diet and early life stress in rats. Physiol Behav. 2022;257:113969. 36181786 10.1016/j.physbeh.2022.113969

[30] Gomez-Pinilla F, Cipolat RP, Royes LFF. Dietary Fructose as a model to explore the influence of peripheral metabolism on brain function and plasticity. Biochim Biophys Acta Mol Basis Dis. 2021;1867(5):166036. 33508421 10.1016/j.bbadis.2020.166036

[31] Bordeleau M, Fernandez de Cossio L, Chakravarty MM, Tremblay ME. From maternal diet to neurodevelopmental disorders: A story of neuroinflammation. Front Cell Neurosci. 2020;14:612705. 33536875 10.3389/fncel.2020.612705 PMC7849357

[32] Souza RT, Mayrink J, Leite DF, Costa ML, Calderon IM, Rocha Filho EA, et al. Metabolomics applied to maternal and perinatal health: a review of new frontiers with a translation potential. Clin (Sao Paulo). 2019;74:e894. 10.6061/clinics/2019/e894 PMC6438130 30916173

[33] Menon R, Jones J, Gunst PR, Kacerovsky M, Fortunato SJ, Saade GR, et al. Amniotic fluid metabolomic analysis in spontaneous preterm birth. Reprod Sci. 2014;21(6):791–803. 24440995 10.1177/1933719113518987 PMC4016728

[34] Tapia G, Suvitaival T, Ahonen L, Lund-Blix NA, Njolstad PR, Joner G, et al. Prediction of type 1 diabetes at birth: cord blood metabolites vs genetic risk score in the Norwegian mother, father, and child cohort. J Clin Endocrinol Metab. 2021;106(10):e4062–71. 34086903 10.1210/clinem/dgab400 PMC8475222

[35] Shearer J, Klein MS, Vogel HJ, Mohammad S, Bainbridge S, Adamo KB. Maternal and cord blood metabolite associations with gestational weight gain and pregnancy health outcomes. J Proteome Res. 2021;20(3):1630–8. 33529033 10.1021/acs.jproteome.0c00854

[36] Guixeres-Esteve T, Ponce-Zanon F, Morales JM, Lurbe E, Alvarez-Pitti J, Monleon D. Impact of maternal weight gain on the newborn metabolome. Metabolites. 2023;13(4). 10.3390/metabo13040561 PMC10142613 37110219

[37] Leon-Aguilar LF, Croyal M, Ferchaud-Roucher V, Huang F, Marchat LA, Barraza-Villarreal A, et al. Maternal obesity leads to long-term altered levels of plasma ceramides in the offspring as revealed by a longitudinal lipidomic study in children. Int J Obes (Lond). 2019;43(6):1231–43. 30568270 10.1038/s41366-018-0291-y

[38] Yeum D, Gilbert-Diamond D, Doherty B, Coker M, Stewart D, Kirchner D et al. Associations of maternal plasma and umbilical cord plasma metabolomics profiles with birth anthropometric measures. Pediatr Res. 2023;94(1):135–42. 10.1038/s41390-022-02449-2 PMC12269892 36627359

[39] Ivorra C, Garcia-Vicent C, Chaves FJ, Monleon D, Morales JM, Lurbe E. Metabolomic profiling in blood from umbilical cords of low birth weight newborns. J Transl Med. 2012;10:142. 22776444 10.1186/1479-5876-10-142 PMC3551816

[40] Wang X, Liu J, Hui X, Song Y. Metabolomics applied to cord serum in preeclampsia newborns: implications for neonatal outcomes. Front Pediatr. 2022;10:869381. 35547553 10.3389/fped.2022.869381 PMC9082809

[41] Hartvigsson O, Barman M, Rabe H, Sandin A, Wold AE, Brunius C, et al. Associations of maternal and infant metabolomes with immune maturation and allergy development at 12 months in the Swedish NICE-cohort. Sci Rep. 2021;11(1):12706. 34135462 10.1038/s41598-021-92239-3 PMC8209090

[42] Ross AB, Barman M, Hartvigsson O, Lundell AC, Savolainen O, Hesselmar B, et al. Umbilical cord blood metabolome differs in relation to delivery mode, birth order and sex, maternal diet and possibly future allergy development in rural children. PLoS ONE. 2021;16(1):e0242978. 33493154 10.1371/journal.pone.0242978 PMC7833224

[43] Ma J, Luo J, He M, Bian X, Li J, Du Y, et al. Umbilical cord blood metabolomics: association with intrauterine hyperglycemia. Pediatr Res. 2022;91(6):1530–5. 33980991 10.1038/s41390-021-01516-4

[44] Moros G, Boutsikou T, Fotakis C, Iliodromiti Z, Sokou R, Katsila T, et al. Insights into intrauterine growth restriction based on maternal and umbilical cord blood metabolomics. Sci Rep. 2021;11(1):7824. 33837233 10.1038/s41598-021-87323-7 PMC8035183

[45] Wang L, Han TL, Luo X, Li S, Young T, Chen C, et al. Metabolic biomarkers of monochorionic twins complicated with selective intrauterine growth restriction in cord plasma and placental tissue. Sci Rep. 2018;8(1):15914. 30374111 10.1038/s41598-018-33788-y PMC6206027

[46] Sanz-Cortes M, Carbajo RJ, Crispi F, Figueras F, Pineda-Lucena A, Gratacos E. Metabolomic profile of umbilical cord blood plasma from early and late intrauterine growth restricted (IUGR) neonates with and without signs of brain vasodilation. PLoS ONE. 2013;8(12):e80121. 24312458 10.1371/journal.pone.0080121 PMC3846503

[47] Cosmi E, Visentin S, Favretto D, Tucci M, Ragazzi E, Viel G, et al. Selective intrauterine growth restriction in monochorionic twin pregnancies: markers of endothelial damage and metabolomic profile. Twin Res Hum Genet. 2013;16(4):816–26. 23701694 10.1017/thg.2013.33

[48] O’Boyle DS, Dunn WB, O’Neill D, Kirwan JA, Broadhurst DI, Hallberg B, et al. Improvement in the prediction of neonatal Hypoxic-Ischemic encephalopathy with the integration of umbilical cord metabolites and current clinical makers. J Pediatr. 2021;229:175–81. e1. 33039387 10.1016/j.jpeds.2020.09.065

[49] Ahearne CE, Denihan NM, Walsh BH, Reinke SN, Kenny LC, Boylan GB, et al. Early cord metabolite index and outcome in perinatal asphyxia and Hypoxic-Ischaemic encephalopathy. Neonatology. 2016;110(4):296–302. 27486995 10.1159/000446556

[50] Reinke SN, Walsh BH, Boylan GB, Sykes BD, Kenny LC, Murray DM, et al. 1H NMR derived metabolomic profile of neonatal asphyxia in umbilical cord serum: implications for hypoxic ischemic encephalopathy. J Proteome Res. 2013;12(9):4230–9. 23931672 10.1021/pr400617m

[51] Walsh BH, Broadhurst DI, Mandal R, Wishart DS, Boylan GB, Kenny LC, et al. The metabolomic profile of umbilical cord blood in neonatal hypoxic ischaemic encephalopathy. PLoS ONE. 2012;7(12):e50520. 23227182 10.1371/journal.pone.0050520 PMC3515614

[52] Sun H, Wang YC, Wang CC, Xu XX, Wang YH, Yan HT, et al. Metabolic profiling of umbilical cord blood in macrosomia. Int J Obes (Lond). 2018;42(4):679–85. 29158542 10.1038/ijo.2017.288

[53] Eguchi A, Sakurai K, Watanabe M, Mori C. Exploration of potential biomarkers and related biological pathways for PCB exposure in maternal and cord serum: A pilot birth cohort study in Chiba, Japan. Environ Int. 2017;102:157–64. 28262321 10.1016/j.envint.2017.02.011

[54] La Torre D, Seppanen-Laakso T, Larsson HE, Hyotylainen T, Ivarsson SA, Lernmark A, et al. Decreased cord-blood phospholipids in young age-at-onset type 1 diabetes. Diabetes. 2013;62(11):3951–6. 23929934 10.2337/db13-0215 PMC3806611

[55] Hashimoto F, Nishiumi S, Miyake O, Takeichi H, Chitose M, Ohtsubo H, et al. Metabolomics analysis of umbilical cord blood clarifies changes in saccharides associated with delivery method. Early Hum Dev. 2013;89(5):315–20. 23178109 10.1016/j.earlhumdev.2012.10.010

[56] Hay WW Jr., Sparks JW. Placental, fetal, and neonatal carbohydrate metabolism. Clin Obstet Gynecol. 1985;28(3):473–85. 3902302 10.1097/00003081-198528030-00003

[57] Abel KM, Dalman C, Svensson AC, Susser E, Dal H, Idring S, et al. Deviance in fetal growth and risk of autism spectrum disorder. Am J Psychiatry. 2013;170(4):391–8. 23545793 10.1176/appi.ajp.2012.12040543

[58] Madsen-Bouterse SA, Romero R, Tarca AL, Kusanovic JP, Espinoza J, Kim CJ, et al. The transcriptome of the fetal inflammatory response syndrome. Am J Reprod Immunol. 2010;63(1):73–92. 20059468 10.1111/j.1600-0897.2009.00791.x PMC3437779

[59] Careaga M, Murai T, Bauman MD. Maternal immune activation and autism spectrum disorder: from rodents to nonhuman and human Primates. Biol Psychiatry. 2017;81(5):391–401. 28137374 10.1016/j.biopsych.2016.10.020 PMC5513502

[60] Che X, Roy A, Bresnahan M, Mjaaland S, Reichborn-Kjennerud T, Magnus P et al. Metabolomic analysis of maternal mid-gestation plasma and cord blood in autism spectrum disorders. Mol Psychiatry. 2023;28(6):2355–69. 10.1038/s41380-023-02051-w 37037873

[61] Schmidt RJ, Liang D, Busgang SA, Curtin P, Giulivi C. Maternal plasma metabolic profile demarcates a role for neuroinflammation in Non-Typical development of children. Metabolites. 2021;11(8). 10.3390/metabo11080545 PMC8400060 34436486

[62] Fanos V, Atzori L, Makarenko K, Melis GB, Ferrazzi E. Metabolomics application in maternal-fetal medicine. Biomed Res Int. 2013;2013:720514. 23841090 10.1155/2013/720514 PMC3690726

[63] Graca G, Goodfellow BJ, Barros AS, Diaz S, Duarte IF, Spagou K, et al. UPLC-MS metabolic profiling of second trimester amniotic fluid and maternal urine and comparison with NMR spectral profiling for the identification of pregnancy disorder biomarkers. Mol Biosyst. 2012;8(4):1243–54. 22294348 10.1039/c2mb05424h

[64] Petrick LM, Arora M, Niedzwiecki MM. Minimally invasive biospecimen collection for exposome research in children’s health. Curr Environ Health Rep. 2020;7(3):198–210. 32535858 10.1007/s40572-020-00277-2 PMC7895307

[65] Denihan NM, Boylan GB, Murray DM. Metabolomic profiling in perinatal asphyxia: a promising new field. Biomed Res Int. 2015;2015:254076. 25802843 10.1155/2015/254076 PMC4329862

[66] Hartvigsson O, Barman M, Savolainen O, Ross AB, Sandin A, Jacobsson B et al. Differences between arterial and venous umbilical cord plasma metabolome and association with parity. Metabolites. 2022;12(2). 10.3390/metabo12020175 PMC8877791 35208249

[67] Emanuele E, Colombo R, Martinelli V, Brondino N, Marini M, Boso M, et al. Elevated urine levels of Bufotenine in patients with autistic spectrum disorders and schizophrenia. Neuro Endocrinol Lett. 2010;31(1):117–21. 20150873

[68] Gagliano A, Murgia F, Capodiferro AM, Tanca MG, Hendren A, Falqui SG, et al. (1)H-NMR-Based metabolomics in autism spectrum disorder and pediatric Acute-Onset neuropsychiatric syndrome. J Clin Med. 2022;11:21. 10.3390/jcm11216493 PMC9658067 36362721

[69] Emwas AH, Roy R, McKay RT, Tenori L, Saccenti E, Gowda GAN et al. NMR spectroscopy for metabolomics research. Metabolites. 2019;9(7). 10.3390/metabo9070123 PMC6680826 31252628

[70] Vivekanandan-Giri A, Byun J, Pennathur S. Chapter five - Quantitative analysis of amino acid oxidation markers by tandem mass spectrometry. In: Conn PM, editor. Methods in enzymology. Volume 491. Academic; 2011. pp. 73–89. 10.1016/B978-0-12-385928-0.00005-5 PMC3159149 21329795

[71] Fiehn O. Metabolomics by gas Chromatography-Mass spectrometry: combined targeted and untargeted profiling. Curr Protoc Mol Biol. 2016;114(4):30. 10.1002/0471142727.mb3004s114 PMC4829120 27038389

[72] Olivier M, Asmis R, Hawkins GA, Howard TD, Cox LA. The need for Multi-Omics biomarker signatures in precision medicine. Int J Mol Sci. 2019;20:19. 10.3390/ijms20194781 PMC6801754 31561483

[73] Picard M, Scott-Boyer MP, Bodein A, Perin O, Droit A. Integration strategies of multi-omics data for machine learning analysis. Comput Struct Biotechnol J. 2021;19:3735–46. 34285775 10.1016/j.csbj.2021.06.030 PMC8258788

[74] Galal A, Talal M, Moustafa A. Applications of machine learning in metabolomics: disease modeling and classification. Front Genet. 2022;13:1017340. 36506316 10.3389/fgene.2022.1017340 PMC9730048

