# Supplementary material for: Disrupted fetal carbohydrate metabolism in children with autism spectrum disorder
Source: J Neurodev Disord. 2025 Mar 29;17:16. doi: 10.1186/s11689-025-09601-z (PMC11954230; doi:10.1186/s11689-025-09601-z)
Supplement: Supplementary file 1 — Supplementary Material 1 [file 11689_2025_9601_MOESM1_ESM.docx]

**Supplementary Table 1. All detected analytes (76 total) given select ion monitoring approach.**

| 2-Hydroxybutyric acid |
| --- |
| 2-Hydroxyglutarate |
| 3-Phosphoglyceric acid |
| cis-Aconitic acid |
| Adenine |
| Adenosine |
| Ribitol |
| L-Alanine |
| 3-Methyl-2-oxovaleric acid |
| Oxoglutaric acid |
| Ketoleucine |
| Alpha-ketoisovaleric acid |
| 2-Aminobenzoic acid |
| Arachidic acid |
| L-Asparagine |
| L-Aspartic acid |
| beta-Alanine |
| 3-Hydroxybutyric acid |
| Cholesterol |
| Citric acid |
| L-Cysteine |
| Cytosine |
| L-Dopa |
| D-Fructose |
| Fructose 6-phosphate |
| Fumaric acid |
| Gamma-Aminobutyric acid |
| D-Glucose |
| Glucose 6-phosphate |
| L-Glutamic acid |
| L-Glutamine |
| Glyceric acid |
| Glycerol |
| Glycine |
| Heptadecanoic acid |
| Heptanoic acid |
| L-Histidine |
| Hypotaurine |
| Indoleacetic acid |
| Indoleacrylic acid |
| Indolelactic acid |
| Indole-3-propionic acid |
| Inosine |
| myo-Inosito |
| Isocitric acid |
| L-Isoleucine |
| L-Kynurenine |
| L-Lactic acid |
| Dodecanoic acid |
| L-Leucine |
| L-Lysine |
| L-Malic acid |
| Malonate |
| D-Mannose |
| L-Methionine |
| Methylmalonic acid |
| N-Acetylserine |
| N-Acetyl-L-tyrosine |
| O-Phosphoethanolamine |
| Palmitic acid |
| L-Phenylalanine |
| Phosphoenolpyruvic acid |
| L-Proline |
| Pyruvic acid |
| D-Ribose |
| D-Sedoheptulose 7-phosphate |
| Serine |
| Serotonin |
| Succinic acid |
| L-Threonine |
| L-Tryptophan |
| L-Tyrosine |
| Uracil |
| Urea |
| L-Valine |
| Xanthine |
